# Shaking Table Attached to Magnetorheological Damper: Simulation and Experiments for Structural Engineering

**DOI:** 10.3390/s22103644

**Published:** 2022-05-10

**Authors:** Alessandro N. Vargas, João G. Raminelli, Marcio A. F. Montezuma, Aldemir Aparecido Cavalini Junior, Ricardo Breganon, Constantin F. Caruntu

**Affiliations:** 1 Electronics Department, Universidade Tecnológica Federal do Paraná, UTFPR, Av. Alberto Carazzai 1640, Cornelio Procópio 86300-000, Brazil; jgraminelli@gmail.com (J.G.R.); montezuma@utfpr.edu.br (M.A.F.M.); 2 Centro de Ciências Exatas e Tecnologia, Faculdade de Engenharia Mecânica, Universidade Federal de Uberlândia, UFU, Av. João Naves de Ávila 2121, Uberlândia 38408-144, Brazil; aacjunior@ufu.br; 3 Instituto Federal do Paraná, IFPR, Av. Dr. Tito 801, Jacarezinho 86400-000, Brazil; ricardo.breganon@ifpr.edu.br; 4 Department of Automatic Control and Applied Informatics, Gheorghe Asachi Technical University of Iasi, Str. Prof. D. Mangeron, No. 26, 700050 Iasi, Romania; caruntuc@ac.tuiasi.ro

**Keywords:** magnetorheological dampers, hysteresis, Dahl model, safe design of structures, shaking table

## Abstract

This paper details how to construct a small-scale shaking table attached to a magnetorheological (MR) damper. The motivation for this construction relies on the increasing interest in modeling the dynamics of MR dampers—MR dampers have been used in structures for safety reasons. To model the MR damper, we use the so-called ‘Dahl model’, which is useful to represent systems with a hysteresis. The Dahl model, validated through experimental data collected in a laboratory, was combined with a linear model to represent a two-story building. This two-story building model allows us to simulate the dynamics of that building when its floors are attached to MR dampers. By doing so, we can assess—through simulation—to what extent MR dampers can protect structures from vibrations. Using data from the ‘El Centro’ earthquake (1940), we can conclude that MR dampers have the potential to reduce the impact of earthquakes upon structures. This finding emphasizes the potential benefits of MR dampers for the safety of structures, which is a conclusion taken from the apparatus detailed in this paper.

## 1. Introduction

Recent developments in magnetorheological (MR) fluid have led to a renewed interest in structural engineering applications, as documented in the literature (e.g., [1,2,3]). When the MR fluid is subjected to a magnetic field, the MR particles align in a format parallel to the field flow lines. This particles’ alignment occurs within milliseconds and affects the fluid viscosity almost immediately [4], even under a low-intensity magnetic field [4,5,6]. Once the particles become aligned, the MR fluid becomes more resistant to any force that tries to move an object floating within the MR fluid. In other words, the MR fluid resistance turns out to be controlled by the magnetic field intensity.

This feature makes the so-called *MR damper* a unique device: controlling the magnetic field intensity of the fluid within the MR damper means controlling the MR damper stiffness. Because of its fast response to changing stiffness, the MR damper has found applications in aircraft landing gear [7], vehicle suspension [8,9,10], cable-stayed bridges [11,12], energy harvesting [13], and rehabilitation robotics [14,15,16].

Over the last few years, an enormous amount of research has been conducted to model MR dampers. Not surprisingly, researchers have pointed out that modeling MR dampers is a difficult task. The difficulty stems from certain MR damper nonlinearities—hard to represent—such as hysteresis [17,18,19], Stribeck effect [20], and Coulomb friction [21]. Even changes in temperature dramatically affect the hysteresis curve in MR dampers; see Figure 2 in [22] for a pictorial illustration. No doubt that the task of modeling MR dampers is a challenge, and it is still an open problem [23]. This fact motivated us to contribute to the modeling of MR dampers, as now detailed.

Research on modeling MR dampers can be split into two categories: static models and dynamical models. As for static models, several researchers have suggested either nonlinear functions or soft-computing techniques. For instance, researchers have utilized the static sigmoid function [20,24], Gompertz function [25], hyperbolic tangent function [26], and neural networks [27,28,29]. These investigations came with a drawback—they require a large database to tune in the corresponding functions.

On the other front, dynamical systems have been effective in modeling real-time MR dampers. Among the different models available in the literature (e.g., [19,30,31,32]), one model in particular has attracted a lot of attention—it is known as the *Bouc–Wen model* (a comprehensive investigation of the Bouc–Wen model is available in the monograph [33]). The Bouc–Wen model has no physical meaning; however, the Bouc–Wen model describes hysteresis, and hysteresis is the phenomenon that drives MR dampers. This assertion finds evidence in many experiments performed over the last two decades; see [3,17,34,35,36,37,38,39] for a brief account. These investigations suggest that the Bouc–Wen model represents the hysteresis behavior of MR dampers. This paper shows experimental data that confirm these investigations (see Section 4).

Recall that the Bouc–Wen model’s parameters define the shape of the hysteresis curve [33]. Yet the authors [34] claim that some of the Bouc—Wen parameters are useless when the task is modeling MR dampers. When removing these particular parameters, the Bouc—Wen model becomes identical to the so-called *Dahl model*. Therefore, the Dahl model is a particular case of the Bouc—Wen model ([21], Section 5).

Using the Dahl model to represent MR dampers also finds support in the literature; see [34,40]. A study has confirmed that the Dahl model can represent large-scale MR dampers with improved performance when the Dahl model accounts for probabilistic uncertainties [30]. Another study expands the Dahl model to incorporate viscous fluid dynamics, thermal expansion properties, and gas compressibility and elasticity [41]. A drawback of the expanded model from [41] is that it results in a large-dimensional nonlinear system, which is difficult to handle from the numerical viewpoint. The authors of another article compare the Dahl model against the different models (i.e., Bouc–Wen, Kwok, Lugre, and algebraic models), showing that the Dahl model is consistent with the data [31]. These investigations suggest that the Dahl model effectively represents the nonlinear dynamics of MR dampers. Here, we show experimental data that support this finding.

The main contribution of this paper is twofold. First, we show how to construct a small-scale shaking table and how to connect it with an MR damper. The motivation for building this shaking table comes from its usefulness in checking how safe the structures are, such as buildings and bridges, when both are subjected to vibrations (see Section 4).

Second, we show experimental data that suggest this small-scale MR damper can be modeled by the Dahl model, which is a finding that is in agreement with other investigations (e.g., [42,43]). The shaking table produced displacements in the MR damper, and the corresponding experimental data were used to identify the Dahl model. It is noteworthy that the Dahl model we have identified produced an error of 21.71% (see Section 4), which is a value in line with the error of 25% reported in [34]. Next, the Dahl model was used in a simulation that illustrates how the MR damper can be deployed to mitigate earthquakes’ effects in a two-story building. These findings set the main contribution of this paper.

### Literature Review on Shaking Table Attached to MR Dampers

The shaking table plays an important role in promoting the safe design of structures. For instance, researchers attach the base of the structure under investigation to the shaking table’s platform and make the shaking table move so that the table imposes certain accelerations upon the base of the platform. Because the researchers can program the shaking table movements, for instance, to reproduce certain earthquake profiles, they can assess the earthquake’s effects upon structures; see the experiments in [44,45,46,47], to name but a few.

When connected to MR dampers, the shaking table expands its usefulness. MR dampers could be attached either to the shaking table’s platform or to the structure itself. By doing so, researchers can assess the role of MR dampers in mitigating the earthquake’s effects on the structure. Several studies have found that MR dampers can drastically reduce accelerations induced by earthquakes; see, for example, the experiment performed for a three-story steel structure [48], three-story steel—concrete structure [49], six-story steel-made building [50], and six-story steel frame structure [51]. This paper contributes to this research topic as well, as clarified next.

**Remark** **1.** *This paper shows how to construct a shaking table attached to an MR damper. The motivation for making this shaking table is to allow us to emulate structures subject to vibrations. When the table oscillates, the MR damper exerts a force that can be controlled through a voltage. Understanding how the position of the table relates to the force and voltage of the MR damper represents the main contribution of this paper.*

In this paper, we use experimental data to obtain the Dahl model. We then use the Dahl model, combined with another model representing a two-story steel structure, in a way that both models characterize the dynamics of that structure under vibrations. To illustrate this model’s effectiveness, we applied accelerations from the ‘El Centro’ earthquake (1940). The corresponding simulated data indicate that the MR damper drastically reduces the earthquake’s overall structure effects. This conclusion supports many experiments performed in the last three decades (e.g., [48,50,52,53,54,55]). In summary, our finding illustrates the potential benefits of our approach to the safe design of structures.

## 2. Experimental Setup

**Figure 1:**
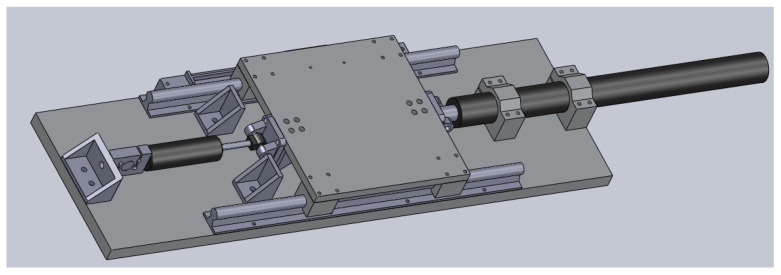
Pictorial representation of the laboratory testbed. The shaking table’s platform is attached to a magnetorheological damper from the left and to an actuator from the right.

This section aims to describe how to construct the shaking table attached to a magnetorheological (MR) damper, as depicted in Figure 1. As can be seen, the shaking table works as a single-axis device. The device has a base with a sliding table over it. On its right side, the sliding table was attached to the shaft of an actuator. According to the actuator’s commands, the shaft moves back and forth along its central axis. The sliding table was attached to an MR damper on its left side. The MR damper, in its other extremity, was attached to a load cell. The load cell measured the force upon the MR damper when the shaking table was working. The shaking table worked with its top platform sliding from left to right and vice-versa, with movements determined by the actuator. A linear encoder was affixed below the sliding platform for measuring the platform displacement.

The data measured in the laboratory were collected according to the next procedure: the electric current generated movements in the actuator shaft, which in turn moved the platform. The force the platform exerted upon the MR damper was measured by the load cell, which was collected through a data acquisition board and recorded on a computer.

**Figure 2:**
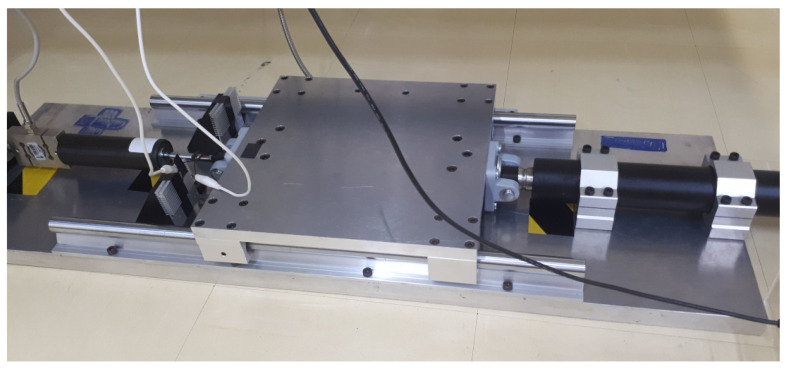
Experimental setup. The shaking table platform moves on rails. The platform has the MR damper attached to the left and the actuator attached to the right.

The experimental setup assembled in the laboratory is shown in Figure 2. The base of the structure was a steel board, which was carefully designed to support the other structural parts. The base had dimensions of 980×300×25.4 mm. Two linear rails were stuck in the base to support over them the sliding platform. The sliding platform was aluminum made with the dimensions of 303×300×15.87 mm. The MR damper used in this study was the MR fluid damper model RD-8041-1, which was manufactured by the Lord Corporation (Cary, NC, USA). The load cell used was the model ZSL, which was manufactured by IWM (São Paulo, Brazil); it has a maximum workload of 500 kg and sensitivity of 1.955 mV/V. The actuator is Glideforce LACT12P-12V-20 Light-Duty Linear Actuator (Las Vegas, NV, USA), which has the form of a ball-screw linear actuator, with the following features: maximum stroke of 457 mm, the gear ratio of 20:1, maximum load speed of 14.3 mm/sec, and maximum current load of 13.2 A. The driver that fed the linear actuator with electricity was the IBT-3 50A H-bridge High-power Motor Drive. This drive received a pulse-width modulation (PWM) signal from an Arduino Uno microcontroller. The linear encoder was manufactured by Sino (Suzhou, Jiangsu, China), model KA-300, with a measuring range of 370 mm and scale precision of 5 μm. The data acquisition board was the model NI PCI-6221 manufactured by National Instruments (Austin, TX, USA).

## 3. Modeling and Identification

Some investigations have concluded that the Dahl model can represent large-scale MR dampers [21,34,40]. This finding motivated us to check whether the Dahl model can also represent small-scale MR dampers. We reach a positive conclusion based on experimental data, as detailed in the sequence.

**Figure 3:**
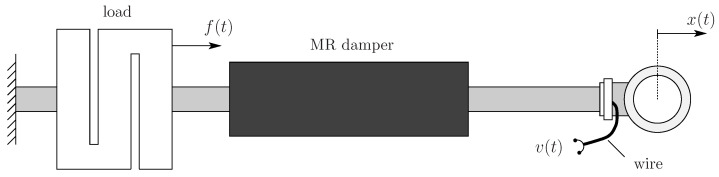
MR damper connected to a load cell. In the system modeling, the voltage v(t) and displacement x(t) represent the two input variables, and the force f(t) represents the output.

From the dynamical systems viewpoint, the MR damper can be interpreted as a two-input one-output system. As input, the MR damper has the displacement of its rod and the voltage applied in its coil. As output, the MR damper has the force exerted upon it [21,34]; see Figure 3.

The Dahl model studied in this paper has been introduced in [21], and it is given by (see [34])

(1) $$\begin{array}{cc} f(t) & =k_{x}\left[v\left(t\right)\right]\dot{x}\left(t\right)+k_{w}\left[v\left(t\right)\right]w\left(t\right),\\ \dot{w}\left(t\right) & =\rho \left[v\left(t\right)\right](\dot{x}\left(t\right)-|\dot{x}\left(t\right)|w\left(t\right)),\;\forall t\geq 0, \end{array}$$

where x(t) denotes the damper rod displacement, v(t) denotes the voltage input of the MR damper, f(t) represents the MR damper force, and w(t) describes the non-physical element that accounts for the damper hysteresis. The parameters $k_{x}$, $k_{w}$, and ρ depend on the voltage v(t) in a nonlinear relation [21,34]. Since these parameters have no physical meaning, they can be taken as polynomials with respect to v(t). The authors of [21,34] have suggested polynomials with degree two; in this paper, we consider the polynomials with degree three, as in [34].

A procedure for the identification of the Dahl model in (1) is recalled next. This procedure is borrowed from [34].

• *Step 1*: Fix some constant u≥0 and set v(t)=u for all t≥0.
• *Step 2*: Move the MR damper in a way that a periodic signal appears in x(t) and collect the corresponding force f(t). Once the MR damper has reached the periodic equilibrium, we consider T>0 as the time necessary to complete a cycle. Separate to the analysis only the data corresponding to the interval [0,T], say (x(τ),f(τ)), τ∈[0,T]. Note that the curve constructed from the pair $\left(\dot{x}\left(\tau \right),f\left(\tau \right)\right)$, τ∈[0,T] must have a hysteresis shape [34].
• *Step 3*: Select the data corresponding to the MR damper loading part, and let the corresponding interval be $\left[0,T^{+}\right]$. Compute
  $$k_{x}\left(u\right)=\frac{f\left(0\right)+f\left(T^{+}\right)}{\dot{x}\left(0\right)+\dot{x}\left(T^{+}\right)}.$$
• *Step 4*: Now consider the system (1) as a function of *x* so as to define the function θ:R↦R as (see [21,34])
  $$\theta \left(x\right)=f\left(x\right)-k_{x}\left(u\right)\dot{x}=k_{w}\left(u\right)w\left(x\right).$$
  From the function θ(x), take some $x_{*}$ such that $\theta (x_{*})=0$ and define the constant
  $$a={\left.\frac{d\theta (x)}{dx}\right|}_{x=x_{*}\;}$$
• *Step 5*: Choose some constant $x_{1,*}>x_{*}$, and calculate
  $$\rho \left(u\right)=\frac{a-{\left.\frac{d\theta (x)}{dx}\right|}_{x=x_{1,*}\;}}{\theta (x_{1,*})}\;\mathrm{and}\;k_{w}\left(u\right)=\frac{a}{\rho (u)}.$$

**Remark** **2.** *The algorithm presented in Steps 1–5 is detailed in [34]. Note that this algorithm follows by simplifying the other algorithm that was developed earlier for the identification of the Bouc—Wen model ([33], Section 5.2). Because the Dahl model is a particular case of the Bouc—Wen model, the algorithm in [34] (i.e., Steps 1–5) is a particular case of the algorithm of ([33], Section 5.2). Next, we present experimental data that emphasize the usefulness of the algorithm in Steps 1–5 for the modeling of a small-scale MR damper.*

The algorithm of Steps 1–5 produces parameters $k_{x}$, $k_{w}$, and ρ that depend on the constant u≥0. For real-time experiments, this algorithm must be repeated several times, evaluating distinct values of *u*. It means that a database containing distinct values of $k_{x}$, $k_{w}$, ρ, and *u*, is created. Using data from a large-scale MR damper, the authors of [34] have created such a database. However, they have simplified it through a third-order polynomial, i.e., they have used the polynomials below to fit the data.

(2) $$\begin{array}{c} k_{x}\left(u\right)=\sum_{i=0}^{3}k_{k,i}u^{i},\;k_{w}\left(u\right)=\sum_{i=0}^{3}k_{w,i}u^{i},\;\rho \left(u\right)=\sum_{i=0}^{3}\rho_{i}u^{i}. \end{array}$$

The approach of [34] motivated us to follow a similar path. Namely, we create a database and simplify it through (2), yet the data were taken from a small-scale MR damper.

## 4. Experimental Results

This section presents the experimental data used in the identification of the Dahl model.

**Figure 4:**
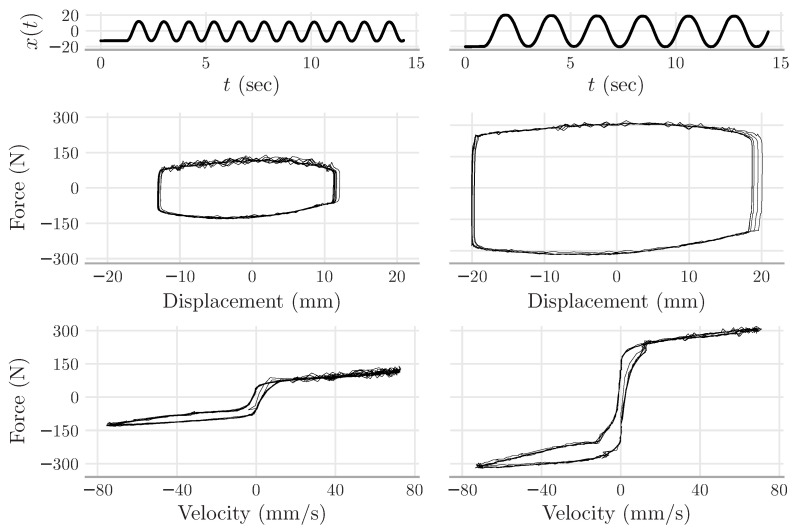
Experimental data. The two upper plots show the displacements made by the shaking table upon the rod of the MR damper. The curves in the left (right) columns correspond to the MR damper with a fixed voltage of u=0 (u=0.88 V). The curves in the second row show that the force induced by displacement increased when the MR damper received the voltage u=0.88 V. The curves in the bottom row show hysteresis, which is a typical phenomenon of MR dampers.

The experiments performed in the laboratory followed the following procedure. The shaking table was programmed to excite the MR damper with displacement inputs as a sine waveform. Sine waves with distinct amplitudes and frequencies were used. Each experiment was performed with a fixed voltage to the MR damper; namely, the MR damper received v(t)=u≥0 during the whole experiment (see Figure 4 for a sample).

The experimental data were then applied in the algorithm of Steps 1–5, which resulted in the database shown in Table 1. We combined the information from this database with a least-square procedure to obtain the third-order polynomials (see (2)).

$$\begin{array}{cc} k_{x}\left(u\right) & =0.085u^{3}+0.034u^{2}+0.493u+0.8,\\ k_{w}\left(u\right) & =3.615u^{3}-24.86u^{2}+201.9u+50.2,\\ \rho (u) & =-2.83u^{3}+17.9u^{2}-39u+42.7. \end{array}$$

By substituting these three polynomials into (1), we simulated the Dahl model for all cases shown in the database of Table 1. In connection with the simulation data, we calculate the error for the experimental data, i.e.,

$$\varepsilon =\frac{∥F_{e}{-F∥}_{1}}{∥F_{e}{∥}_{1}},$$

where ${∥\;\cdot \;∥}_{1}$ stands for the usual L1-norm, $F_{e}$ denotes the experimental force measured in the laboratory for all experiments, and *F* denotes the corresponding Dahl model’s force.

**Figure 5:**
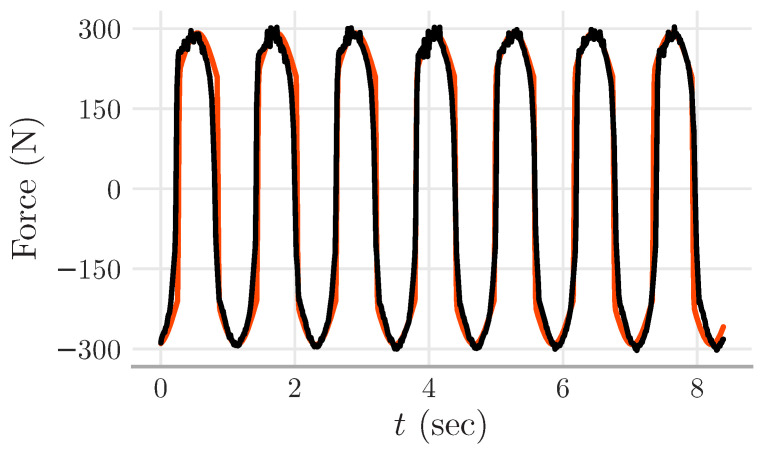
Simulation and experimental data from the MR damper. The simulated curve (red) was obtained from the Dahl model. The experimental curve (black) corresponds to the MR damper with a voltage of 1.98 V, an amplitude of 9.59 mm, and a frequency of 0.8378 Hz. The simulated curve seems to approximate the experimental curve (error of 13.39%).

For the sake of illustration, Figure 5 shows part of the experimental data and their simulation counterpart. Note that both curves seem to fit, i.e., the error from this piece of data shown in Figure 5 was 13.39%.

The error obtained from all experimental and simulation data is 21.71%—this error approximates the error of 25% reported in [34]. This experimental finding suggests that the Dahl model be able to represent a small-scale MR damper, which is evidence that complements the studies in [42,43] that have suggested the Dahl model can represent large-scale MR dampers.

### 4.1. Limitations

This study has some modeling limitations, as described next.

Modeling MR dampers is an open problem. As the experiment indicates, the Dahl model seems able to represent a small-scale MR damper; however, the Dahl model yields a significant modeling error. This modeling error is compatible with what is known in the literature, considering that the different models available so far give rise to error values of up to 25% (cf., [1,34,54,56]). According to researchers, modeling errors arise when the system attempts to model hysteresis [17,18], Stribeck effect [20], and Coulomb friction [21]. The pulse-like motion also affects the nonlinear behavior of dampers [57]. Not to mention that temperature changes drastically affect hysteresis ([22], Figure 2), which complicates even more the modeling task. Even soft-computing-based models suffer noticeable modeling errors [27,35,37]. These facts combined confirm how difficult the task of modeling MR dampers is.

In summary, the Dahl model presented here has a clear, practical benefit—it has few parameters that can be easily identified through Steps 1–5; see Remark 2 in connection. The next section describes how the Dahl model can be used in simulating structures subject to earthquakes, which illustrates the potential of MR dampers for the safety of structures.

### 4.2. Motivation for Earthquake Simulation

It is usual to see building structures collapse during earthquakes [58]. To make these structures safer, researchers have deployed different kinds of energy dissipation devices ([59], Section 7.10), ref. [60]. Among those devices, one—in particular—has drawn the researchers’ attention—the MR damper. Researchers have relied on shaking table experiments to learn how MR dampers can be used to mitigate the effects of earthquakes. For instance, the authors of [53] conducted a shaking-table test on a three-story steel structure, showing the benefits of attaching an MR damper to the building’s first floor. Subsequent studies have found similar results when testing many small-scale structures, such as those in the format of three-story steel structures [48,55], three-story steel–concrete structures [49], and six-story steel frame structures [50,51]. A common feature in these investigations is that the MR damper drastically reduced the earthquake’s effects on the overall structure. This section provides a contribution that supports these findings.

**Figure 6:**
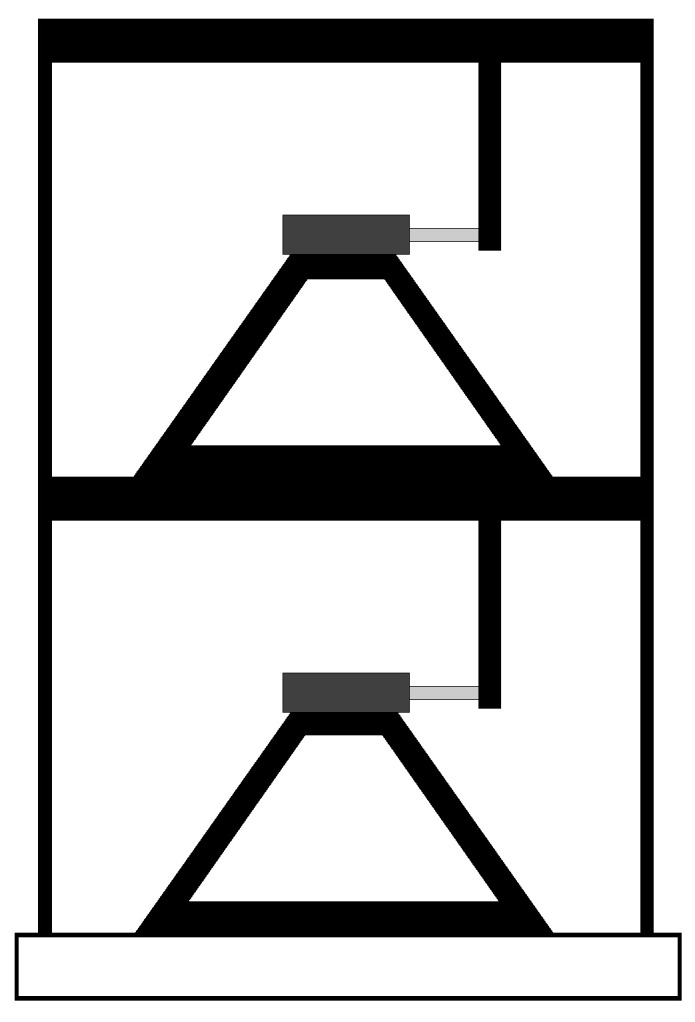
Diagram of the two-story shear structure. The MR dampers are identical and are attached to both floors. The floors are allowed to move only in the horizontal direction.

The main contribution of this section is to illustrate the benefits of MR dampers for structures. To do so, we simulate the structure of Figure 6, which comprises a two-story shear structure with MR dampers attached to both floors. The idea that motivates this simulation stems from [53], which describes the benefits of an MR damper attached to the building’s first floor. In this paper, the two-story structure of Figure 6 was simulated through vibrations from the ‘El-Centro’ earthquake (1940). As the simulation data indicate, the MR dampers dramatically reduce the vibrations from the ‘El-Centro’ earthquake upon the two-story structure. Although based only on simulation, our conclusion coincides with experiments that have been documented in the literature (e.g., [48,50,52,53,54,55]).

In summary, the findings of this section illustrate the potential benefits of our approach to the safe design of structures.

### 4.3. Simulation Results

Consider the two-story shear building depicted in Figure 6. The modeling and simulation of this building are presented next. For this purpose, consider $x_{1}$ and $x_{2}$ as the first and second floor displacements, respectively. Define the function $f:R^{2}\mapsto R$ as the force generated by the Dahl model in (1). The Dahl model’s input variables are the displacements $x_{i}$, i=1,2, and voltage for the MR damper *v*, and the corresponding output is the force $f(x_{i},v)$.

The next two equations define the motion dynamics of the two-story shear building (e.g., [61], Section 8.1, p. 214):

(3) $$\begin{array}{cc} m_{1}{\ddot{x}}_{1}+c_{1}{\dot{x}}_{1}+k_{1}x_{1}-c_{2}\left({\dot{x}}_{2}-{\dot{x}}_{1}\right)-k_{2}\left(x_{2}-x_{1}\right) & =-m_{1}{\ddot{x}}_{g}-f\left(x_{1},v\right),\\ m_{2}{\ddot{x}}_{2}+c_{2}\left({\dot{x}}_{2}-{\dot{x}}_{1}\right)+k_{2}\left(x_{2}-x_{1}\right) & =-m_{2}{\ddot{x}}_{g}-f\left(x_{2},v\right), \end{array}$$

where ${\ddot{x}}_{g}$ denotes the ground acceleration, and the constants $m_{i}$, $c_{i}$, and $k_{i}$, i=1,2, correspond to the masses, damping coefficients, and total stiffness of the columns, respectively. The model in (3) is valid only if the floors of Figure 6 do not bend. In other words, the damper rods move only in the horizontal direction. This assumption makes the simulation simpler, as suggested in [53].

For the simulation of (3), we borrow the parameters from the two-story steel building identified in [62]. The parameters are $m_{1}=m_{2}=24.3$ (N$s^{2}$/m), $c_{1}=0.939$, $c_{2}=0.29$ (Ns/m), and $k_{1}=0.682\times 10^{4}$, $k_{2}=0.822\times 10^{4}$ (N/m). In addition, the ground acceleration ${\ddot{x}}_{g}$ was considered, which was taken from the El Centro earthquake and resized by a scale factor of 10% upon its amplitude. In fact, we scaled down the amplitude of the El Centro earthquake by a 10% factor so as to allow the corresponding accelerations to cope with the MR damper displacements.

The building dynamics were investigated under three distinct cases: (i) building without MR damper; (ii) building with MR damper in the *passive-off* mode (i.e., v(t)≡0 V); and (iii) building with MR damper in the *passive-on* mode (i.e., v(t)≡1.98 V).

**Figure 7:**
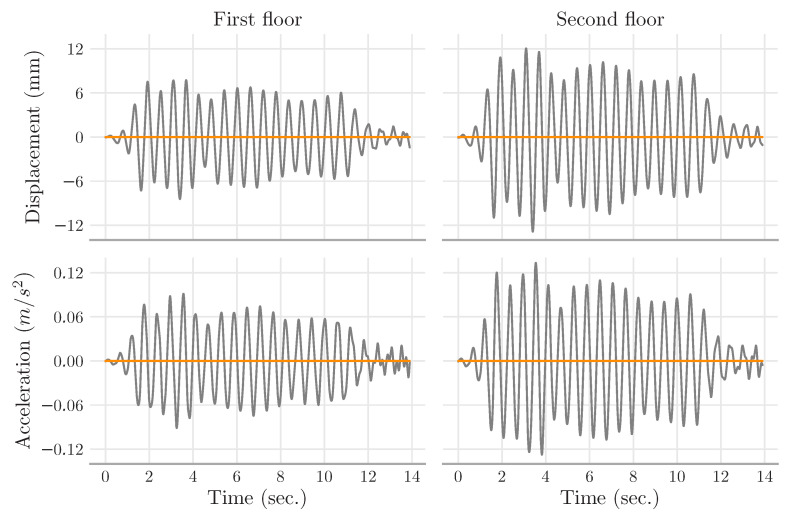
Simulation data for the two-story shear building of Figure 6. The ground of the building was subjected to the accelerations corresponding to 10% of the amplitude of the El Centro earthquake. The building’s dynamics with and without the MR damper are depicted in orange and gray, respectively. The MR damper is in ‘passive-on’ mode. The data indicate that the MR dampers wipe out the vibrations on both floors.

The simulation data indicate that the peak values of displacements and accelerations upon the floors diminish drastically when the MR damper remains attached to the building; see Table 2. For illustration, the building dynamics with and without the MR damper are illustrated in Figure 7 only for the first fourteen seconds (the ‘El Centro’ earthquake lasted around fifty seconds). What stands out in Figure 7 is the dramatic decline in vibrations—the MR dampers wipe out the earthquake’s effects. This simulation confirms the experiments documented in [48,50,52,53,54,55].

To sum up, because the MR dampers decreased vibrations in the building, we can conclude that attaching them to the floors is a way to improve the building’s safety.

**Remark** **3.** *The data and source code used in the analysis made in this manuscript are freely available on GitHub https://github.com/labcontrol-data/mr-damper (accessed on 28 April 2022) and archived in Zenodo [63]. The documents detailing the construction of the shaking table attached to the MR-damper are also freely available.*

## 5. Concluding Remarks

This paper has detailed the construction of a functional, small-scale shaking table. The shaking table was attached to a magnetorheological (MR) damper, finding the motivation to design safer structures. Constructing the shaking table attached to the MR damper, together with the identification of the MR damper, represents the main contribution of this paper.

Experiments were performed for the shaking table in a way that the shaking table produced displacements in the MR damper rod. The displacements were taken under distinct frequencies and amplitudes. The corresponding data were then used to identify the Dahl model—the Dahl model is able to represent the hysteresis of the MR damper as documented in [21,34]. The Dahl model represents the MR damper’s input–output relation: displacement (input) and force (output).

Real-time experiments taken from both the shaking table and the MR damper were used to feed the Dahl model. Next, this model was used to simulate an earthquake upon a two-story steel building (see Figure 6). In the simulation, two MR dampers were attached to two floors to mitigate the effects of the ‘El Centro’ earthquake. The Dahl model represented the MR dampers in the simulation. According to the simulation data, the MR dampers drastically reduced the El Centro earthquake’s effects on the building. This finding corroborates the literature that supports using MR dampers to improve the safety of structures [48,49,50,53,55].

## Figures and Tables

**Table 1:** Data correspond to experiments that were carried out in the laboratory. The values of $k_{x}$, $k_{w}$, and ρ are given by the algorithm of Steps 1–5.

Input (V)	Amplit. (mm)	Frequency (Hz)	$k_{x}$	$k_{w}$	ρ
0	24.75	0.8403	0.7682	57.42	12.375
0	11.40	0.8696	0.6835	59.46	30.57
0	10.86	0.8932	0.7144	56.55	34.14
0	9.98	0.9434	0.7040	53.48	39.20
0	9.13	0.9709	0.8836	48.80	66.03
0	8.13	1.0100	0.7409	49.18	44.04
0	7.58	1.0530	0.7716	50.26	40.07
0	6.82	1.0990	0.9868	38.01	57.50
0	6.14	1.1490	0.8451	46.16	45.67
0	5.62	1.2200	0.9223	43.02	40.67
0.88	19.38	0.4629	1.0640	236.63	19.42
0.88	17.94	0.4808	1.2243	219.63	20.02
0.88	16.43	0.4926	1.2442	224.25	19.16
0.88	13.45	0.5525	1.5333	200.23	18.90
0.88	14.88	0.5236	1.2591	210.33	20.80
0.88	9.86	0.6250	0.8947	225.61	19.54
0.88	12.34	0.5682	1.4875	197.94	22.24
0.88	11.05	0.5988	1.3647	203.51	19.54
0.88	8.79	0.6667	1.4000	193.90	22.68
0.88	11.21	0.8474	1.6689	184.56	21.96
1.98	9.59	0.8378	2.2698	412.48	10.95
1.98	8.99	0.8671	2.0742	403.59	12.94
1.98	8.28	0.9010	2.4235	393.54	11.07
1.98	7.55	0.9438	2.4632	381.02	11.88
1.98	6.72	0.9688	2.1390	398.57	10.17
1.98	6.18	1.0190	3.0239	360.94	18.26
1.98	5.54	1.0520	2.7250	366.70	20.52
1.98	5.01	1.1080	2.8490	357.63	11.20
1.98	4.34	1.1710	2.7943	359.09	10.80
1.98	3.81	1.2050	3.0000	372.50	17.48

**Table 2:** Absolute peak values registered in the simulation data.

Variable (Unity)	No MR Damper	Passive-Off	Passive-On
$x_{1}$ (mm)	8.4299	0.00492	0.001864
$x_{2}$ (mm)	12.8628	0.00502	0.001867
${\ddot{x}}_{1}$ (m/s) $\times \;10^{-4}$	913.78	3.65	1.124
${\ddot{x}}_{2}$ (m/s) $\times \;10^{-4}$	1334.47	3.97	1.126

## Data Availability

Data supporting reported results can be supplied by the authors.
